# How Do Countries’ Health Information Systems Perform in Assessing Asylum Seekers’ Health Situation? Developing a Health Information Assessment Tool on Asylum Seekers (HIATUS) and Piloting It in Two European Countries

**DOI:** 10.3390/ijerph14080894

**Published:** 2017-08-08

**Authors:** Kayvan Bozorgmehr, Simone Goosen, Amir Mohsenpour, Anna Kuehne, Oliver Razum, Anton E. Kunst

**Affiliations:** 1 Department of General Practice and Health Services Research, University Hospital Heidelberg, 69120 Heidelberg, Germany; mohsenpour@stud.uni-heidelberg.de; 2 Netherlands Association of Community Health Services, 3524 SJ Utrecht, The Netherlands; sgoosen@ggdghor.nl; 3 Health Collective Berlin, 12053 Berlin, Germany; kuehne.a@posteo.net; 4 Department of Epidemiology & International Public Health, School of Public Health, Bielefeld University, 33501 Bielefeld, Germany; oliver.razum@uni-bielefeld.de; 5 Department of Public Health and Epidemiology, Academic Medical Centre, 1105 AZ Amsterdam, The Netherlands; a.kunst@amc.uva.nl

**Keywords:** health information system, health systems research, health systems strengthening, asylum seeker, refugee, health inequality, performance, Germany, The Netherlands

## Abstract

*Background:* Accurate data on the health status, health behaviour and access to health care of asylum seekers is essential, but such data is lacking in many European countries. We hence aimed to: (a) develop and pilot-test an instrument that can be used to compare and benchmark the country health information systems (HIS) with respect to the ability to assess the health status and health care situation of asylum seekers and (b) present the results of that pilot for The Netherlands (NL) and Germany (DE). *Materials and Methods*: Reviewing and adapting existing tools, we developed a Health Information Assessment Tool on Asylum Seekers (HIATUS) with 50 items to assess HIS performance across three dimensions: (1) availability and detail of data across potential data sources; (2) HIS resources and monitoring capacity; (3) general coverage and timeliness of publications on selected indicators. We piloted HIATUS by applying the tool to the HIS in DE and NL. Two raters per country independently assessed the performance of country HIS and the inter-rater reliability was analysed by Pearson’s rho and the intra-class correlation (ICC). We then applied a consensus-based group rating to obtain the final ratings which were transformed into a weighted summary score (range: 0–97). We assessed HIS performance by calculating total and domain-specific HIATUS scores by country as well as absolute and relative gaps in scores within and between countries. *Results*: In the independent rating, Pearson’s rho was 0.14 (NL) and 0.30 (DE), the ICC yielded an estimated reliability of 0.29 (NL) and 0.83 (DE) respectively. In the final consensus-based rating, the total HIATUS score was 47 in NL and 15 in DE, translating into a relative gap in HIS capacity of 52% (NL) and 85% (DE) respectively. Shortfalls in HIS capacity in both countries relate to the areas of HIS coordination, planning and policies, and to limited coverage of specific indicators such as self-reported health, mental health, socio-economic status and health behaviour. The relative gap in the HIATUS component “data sources and availability” was much higher in Germany (92%) than in NL (28%). *Conclusions*: The standardised tool (HIATUS) proved useful for assessment of country HIS performance in two countries by consensus-based rating. HIATUS revealed substantial limitations in HIS capacity to assess the health situation of asylum seekers in both countries. The tool allowed for between-country comparisons, revealing that capacities were lower in DE relative to NL. Monitoring and benchmarking gaps in HIS capacity in further European countries can help to strengthen HIS in the future.

## 1. Introduction

In 2015, the European Union (EU) received the largest number of forcibly displaced migrants since World War II: over 1.2 million asylum seekers were registered [1]. Forced migration can represent a challenge to transit and destination countries’ health systems in terms of rapid humanitarian responses as well as provision of high-quality care in line with concepts of universal health coverage [2]. Meeting these challenges requires strong health information systems (HIS) which include migrant-sensitive data to produce valid and timely information on health status and health care needs. Data on the short- and long-term health situation during the asylum process—that can take many years—is essential especially for policy makers, health planners and public health professionals at national, regional and local levels. HIS data need to be appropriately disaggregated [3,4,5] so that systematic disparities in health (or its determinants) between social groups [6] can be detected and the impact of policies on health equity and access to care be measured. However, such data is scarce [7,8] and asylum seekers stuck in their asylum process do not appear or cannot be recognised as such in any health register for years.

To improve HIS it is important to monitor their performance and have benchmarks against which countries can identify their relative strengths and weaknesses. The WHO recommends the HIS performance index (HIS-PIX) for the assessment of country HIS. This tool assesses country capacity to collect relevant data at appropriate intervals, periodicity, timeliness, contents of data collection tools and availability of data on key indicators, as well as country capacities for synthesis, analysis and validation of data [9]. HIS-PIX is, however, focussed on low- and middle-income countries, and is applicable to national systems and populations rather than to inequalities within populations.

We hence aimed to: (a) develop an instrument that can be used to compare and benchmark the country HIS with respect to the ability to assess the health status and health care situation of asylum seekers; and (b) pilot this instrument by applying it to The Netherlands and Germany.

## 2. Materials and Methods

### 2.1. Development Process of the Assessment Tool

The development of the tool was guided by the framework of the Health Metrics Network (HMN) of the World Health Organization (WHO) [10], and by guidelines for monitoring health inequalities in the EU [11]. We adapted relevant dimensions and indicators of the HMN framework [10] and the HIS-PIX tool [9] to suit our objectives of measuring country ability to assess the health status and health care situation of asylum seekers. An asylum seeker was considered a person who has applied for recognition as refugee in a country other than his/her own, and is awaiting decision on his/her application. We formulated new items, piloted preliminary versions, and performed revisions in several rounds of written and oral feedback as well as face-to-face discussions until consensus on dimensions, subscales, items, and response options was reached. The dimensions and indicators were chosen based on HISPIX, dimensions of HIS that were found in the literature, an overview of health issues particularly relevant for refugee population, and authors’ practical experience in monitoring the health of this population.

The final tool—a Health Information Assessment Tool on Asylum Seekers (which we named HIATUS, version 1.0)—consisted of three dimensions, 12 subscales and a total of 50 items (Table 1).

### 2.2. Dimensions and Sub-Scales of the Tool

HIATUS assesses HIS performance across three dimensions of country HIS:

(D1) Data sources and data availability: measures the *availability* of data related to asylum seekers across HIS data sources as well as the *extent of details available* across five subscales.
(D2) Resources and capacity: measures *HIS resources* and *(monitoring) capacity* focusing on the areas of coordination, planning and policies related to health monitoring in asylum seekers.
(D3) Published indicators and reports: measures the *general coverage* and *timeliness* of published information on selected key indicators across six sub-scales (self-reported health indicators, non-communicable diseases, infectious diseases, mental health, socio-economic position, and health-related behaviours) in the last 10 years.

HIATUS comprises 50 items, each rated on an ordinal scale (yes/limited/no) including a residual category (don’t know) to assess data availability across relevant data sources, HIS resources and capacity, as well as coverage and timely publication on key indicators (Table 1). For each item, we formulated specific criteria to define a rating as ‘limited’. HIATUS is meant to be primarily applied at national levels, but it might be applied to assess the HIS at subnational levels following the same procedure.

Answer options are scored as 0 (no), 1 (limited) or 2 (yes) scores, and weighted and then summed to receive the final HIATUS score. We weighted the subscale “D1.5—Microdata” availability by the factor 0.5 due to the comparably large number of items in this subscale, which would give a high emphasise on microdata availability without weights (i.e., if all items of the tool were weighted equal). We further weighted the items in dimension “D2—HIS resources & capacity” by the factor 2 to account for the importance of this dimension and compensate for the comparably small number of items, which would lead to a lower contribution to the overall score without weights. The final weighted HIATUS score thus ranged between 0 (minimum score) and 97 (maximum score).

### 2.3. Pilot Test and Cross-Country Comparison

We then applied HIATUS (version 1.0) to the HIS in Germany (DE) and The Netherlands (NL) to measure and compare country ability to assess the health situation of asylum seekers. In a first step, two raters per country with expertise in health information systems and knowledge on the data situation related to health of asylum seekers independently assessed the performance of country HIS (January–April 2016). We then assessed inter-rater reliability by means of Pearson’s rho and the intra-class correlation (ICC).

Additionally, we performed a consensus-based rating instead of an independent rating (July–August 2016). In Germany, we involved a third rater who was not involved in the development process. Selection criteria for the third rater were again expertise in health information systems and knowledge on the data situation related to health of asylum seekers. Based on the consensus-based rating, we calculated dimension-specific and total HIATUS scores by country to reflect HIS capacity. To assess and compare country performance, we calculated the following types of measures:

(1) Absolute gaps in capacity *within* countries: the difference between achieved HIATUS score of a given country and the maximum achievable HIATUS score.
(2) Relative gaps in capacity within countries: calculated as the absolute gap relative to the maximum achievable HIATUS score
(3) Absolute gaps *between* countries: the difference in HIATUS scores between two countries in absolute terms.

## 3. Results

In the independent rating, Pearson’s rho was 0.144 (NL) and 0.30 (DE), the ICC yielded an estimated reliability of 0.290 (NL) and 0.830 (DE) respectively (Table 2).

**Figure 1:**
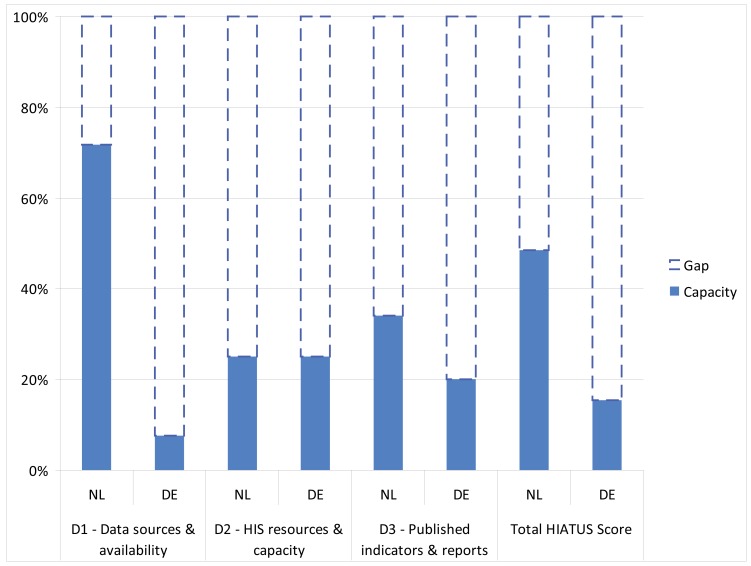
HIS performance as measured by HIATUS, by country and dimension. Y-Axis: Percentage of total HIATUS scores achieved (capacity) and relative within-country difference to maximum achievable score (gap).

In the consensus-based rating, the total HIATUS score was 47 in NL and 15 in DE, translating into a relative within-country gap in HIS capacity of 52% (NL) and 85% (DE) respectively (Figure 1).

With the exception of the dimension of HIS resources and capacity (D2), HIS performance was higher across all HIATUS dimensions in NL compared to DE (Figure 1), resulting in 32-score absolute difference in capacity between countries (Table 3).

The within-country gap in data availability across relevant sources (relative to the maximum achievable score) was much higher in Germany (92%) than in NL (28%). Shortfalls in HIS capacity in both countries relate to the areas of HIS coordination, planning and policies (75% gap in DE/NL respectively), and to limited capacity regarding coverage of specific indicators such as self-reported health (DE: 12.5% and NL: 25% capacity), non-communicable diseases (DE: 0% and NL: 25% capacity) socio-economic status (DE: 30% and NL: 17% capacity) and health behaviour (DE: 0% and NL: 37.5% capacity). There was also a lack of individual-level data in data sources in Germany available to researchers or policy makers in which asylum seekers are both represented and identifiable. Further details on the HIATUS dimensions and subscales as well as the weighted maximum scoring per dimension are listed in Table 3.

## 4. Discussion

HIATUS is a tool that helps countries to assess their HIS with respect to the ability to assess the health status and health care access situation of asylum seekers. It allows for cross-country comparison and benchmarking of HIS performance in this area. We developed the tool as a generic tool to assess the HIS of any country with a well-developed health system. While there is considerable heterogeneity in country HIS, a comparative assessment can help countries to assess their relative strengths and weaknesses, and to identify “benchmark” countries that illustrate how the HIS of one’s own country could perform better. As part of a piloting, we applied the tool to country HIS in DE and NL and compared the performance of the two countries.

The tool proved to be best applicable in a team of at least two persons, in which ratings are performed based on consensus. There are two main reasons for that. Firstly, scoring with HIATUS is based on subjective considerations, which can be expressed, discussed and weighted in teams. Our experience is that team-based scoring is thus important to arrive at reasoned, consensus-based scoring. This also illustrates that the HIATUS instrument will need to be more explicit on the criteria to be applied in future versions. Secondly, the tool covers a wide range of topics in different areas of country HIS, and no single expert will realistically be knowledgeable about all aspects. Moreover, in federal countries such as Germany there may be differences between individual states. Combining the expertise of different public health professionals proved to yield a meaningful and insightful picture of the respective HIS performance. Based on our practical experience of using the tool, a higher number of raters per country may be helpful provided that they are equipped with complementary expertise in HIS, health data collection and refugee issues.

The assessment and cross-country comparison revealed several strengths and weaknesses in the HIS of respective countries. Germany hosts the largest numbers of asylum seekers in the EU. With a capacity of less than 20% of the maximum possible total HIATUS score, there is considerable space for strengthening and improving the HIS in Germany in this area. In contrast, the HIS in the Netherlands performed better, as measured by the HIATUS score, especially with respect to the availability of data across data sources. This is also reflected in the existence of a wide range of epidemiological studies that contributed to the evidence base on health among asylum seekers and that were used to influence health policies (e.g., relocations of children of asylum seekers [12], free provision of contraceptives [13]) and in targeted health promotion activities (e.g., increased drowning risk, high prevalence of diabetes) [14]. The explanation for the huge difference between the two countries is mainly explained by the fact that The Netherlands has established a system of health care registries and notification systems, and was able to include the group of asylum seekers into these data sources. In contrast, existing data sources in Germany are yet less developed and those who exist are often unable to stratify by migrant group or asylum seeker status.

A full HIATUS score reflects a situation in which a HIS is perfectly able to provide data on respective indicators and stratify in existing data sources according to asylum seeker status. It may reasonably be argued that a full score is unlikely to be achieved even for countries with a highly developed health system and a strong HIS. However, we think that the added value of the tool is to depict the gap between the real capacity of country HIS and an “ideal” situation. There may be good reasons to strive for sub-optimal HIS rather than a perfect HIS (e.g., trade-offs between investments in HIS and other blocks of the health system). The value of HIATUS, and its international application, would be to provide the data to inform such a choice.

The HIATUS may also be used for assessing the HIS regarding the host or national population. Overall HIS performance would serve as another benchmark, and help to assess how much improving the refugee HIS would depend on making particular efforts for the refugee populations versus addressing fundamental HIS limitations for the country at large. Further research should therefore assess how the approach taken in HIATUS can be applied to measure general HIS performance or specific performance for other population groups.

Strengthening a HIS requires intellectual, political and financial investments. Investments directed to HIS performance also contribute to strengthening of the overall health system, as other blocks of a health system (service provision, financing, health workforce, and governance) rely on the availability of valid, reliable and timely data. Good access to comprehensive health care can also facilitate data availability, especially from medical records sources. However, when access to health care is not given or only limited in scope, targeted studies and signals from health professionals and others working with asylum seekers become more important to capture potential gaps between health needs and access, e.g., for vulnerable sub-groups among asylum seekers, pointing to areas where the right to health is not fulfilled [3,4].

With HIATUS, we provide a tool for countries to assess their own HIS in an area of international importance related to asylum seekers health and health care. HIATUS proved to have a moderate to low reliability in individual rating, so that a consensus-based rating is the recommended approach to assess country HIS when using the tool. Further studies are needed to assess test-retest reliability of the team based rating, or inter-rater reliability of ratings for the same country between different teams. Further requirements of a valid assessment tool, such as sensitivity to change, still need to be explored in future studies using HIATUS. As country HIS are subject to constant or dynamic changes, a country’s HIS should be assessed using HIATUS at least every 5 years, but also after major changes to the HIS have been implemented.

Further refinements may be applied to the weighting of items to better reflect their relative importance. The applied weights were in fact chosen arbitrarily in a manner that reflected the views of the authors. However, weightings could be applied to items e.g. based on prevalence estimates of predominant conditions, size of special population groups in respective countries (e.g., minors), or particularities of the asylum process. Such weights could also be derived more systematically based on methods to capture the views of experts in the field of health and health care among asylum seekers. If the need arises in a country where HIATUS is used, further dimensions and indicators could be added or amended. Further relevant indicators might refer to substance dependence, population coverage of services, and unmet health care needs or barriers to obtaining care.

A European Union-wide upscale of the performed cross-country comparison would form an ideal opportunity to further test and refine the tool in future studies. This would support objective evaluation of country HIS, enable countries to take measures directed to HIS strengthening, and facilitate international exchange about challenges, strategies, and solutions to monitor the health of asylum seekers across the EU.

## 5. Conclusions

HIATUS proved useful to assess the performance of country HIS regarding the health situation of asylum seekers in two EU countries. Consensus-based rating turned out to be preferable due to the low to moderate reliability of independent rating. The standardised tool revealed substantial limitations in HIS performance in both countries, especially in DE. We recommend the use of HIATUS for countries to assess, and then if necessary improve, the performance of their HIS with regard to asylum seekers. HIATUS should be applied to a larger number of EU countries in order to compare and benchmark HIS performance. An upscale of the assessment to include further EU countries could help to reveal common gaps in and may help to formulate joint strategies to strengthen HIS capacity and ultimately to improve access to healthcare for asylum seekers in Europe.

## Figures and Tables

**Table 1:** Dimensions, sub-scales and items of HIATUS (version 1.0).

**D1—Data Sources & Availability**
**D1.1—Population based records**
Are asylum seekers represented and identifiable in:
the population registry?
population census or demographic data obtained by census-like approaches?
the death registry? Can a distinction be made by cause of death?
nationally representative health interview surveys?
**D1.2—Health records**
Are asylum seekers represented and identifiable in:
claims data or other service utilisation data based on (routine or specific) registries in the health system?
any disease registers for specific non-communicable diseases?
notification-systems for infectious diseases? Is it possible to obtain information on denominator data to calculate incidence or prevalence rates?
**D1.3—Sub-group specific records**
Are there records that allow assessing the health status of or access to health care for:
victims of torture or violence?
unaccompanied minors?
pregnant women?
accompanied children/minors?
**D1.4—Resource records**
Are data available on:
the volume of health facilities and key health services specifically provided to asylum seekers (e.g., number, size, distribution)?
human resources for health specifically concerned with asylum seekers (e.g., density, composition and distribution)?
financing and expenditure for health services specifically provided to asylum seekers?
**D1.5—Microdata**
Are microdata (i.e., individual level data) practically available for researchers or policy makers (e.g., upon request) from:
population registry?
death registry?
population surveys, e.g., health interview surveys?
claims data or other routine data of the health care system?
disease registers?
infectious disease notification systems?
records on resources and health services inputs?
**D2—HIS Resources & Capacity**
**D2.1 Coordination, Planning and Policies**
Is there a:
written plan in active use to comprehensively monitor the health status or health care access of asylum seekers?
functioning national organisation responsible for coordination, planning and implementation of HIS for asylum seekers?
**D3—Published Indicators and Reports**
**D3.1—Self-reported health**
Are there any published indicators, statistics or reports of studies covering self-reported health indicators in the last 10 years such as:
self-rated general health (from poor to very good)?
self-reported access to health care services?
self-reported impairments of disabilities?
self-reported longstanding chronic illnesses?
**D3.2—Non-communicable diseases**
Are there any published indicators, statistics or reports of studies covering non-communicable diseases in the last 10 years such as:
cardiovascular diseases (e.g., stroke, ischemic diseases, myocardial infarction, angina pectoris or heart failure)?
diabetes?
obesity/overweight or under-nutrition?
cancer types?
musculoskeletal diseases?
accidents and injuries (excluding suicidal behaviour)?
**D3.3—Infectious diseases**
Are there any published indicators, statistics or reports of studies covering infectious diseases in the last 10 years such as:
tuberculosis?
HIV/AIDS?
hepatitis B or C?
vaccine preventable diseases?
**D3.4—Mental health**
Are there any published indicators, statistics or reports of studies covering mental health conditions in the last 10 years such as:
depression (including depressive symptoms)?
anxiety disorders?
post-traumatic stress disorder (PTSD)?
suicidal behaviour including death from suicide?
**D3.5—Socio-economic status**
Are there any published statistics or reports covering any indicators of socio-economic status in the last 10 years such as:
level of educational achievement of adult asylum seekers?
employment and type of occupation among asylum seekers?
income, welfare transfers or poverty among asylum seekers?
**D3.6—Health behaviour**
Are there any published indicators, statistics or reports covering health-related behaviours in the last 10 years such as:
alcohol intake (amount, frequency)?
smoking (current status, amount)?
physical activity (type of activity, amount)?
unsafe sex?

**Table 2:** Inter-item correlations, intra-class correlation and inter-rater reliability.

Measure	Country					
	The Netherlands			Germany		
**Inter-item correlations by dimension**	*rho*	*p*-value	*N*	*rho*	*p*-value	*N*
D1—Data sources & availability	0.257	0.237	23	0.510	0.013	23
D2—HIS resources & capacity *	-	-	2	-	-	2
D3—Published indicators & reports	0.066	0.754	25	0.256	0.217	25
All HIATUS Items	0.144	0.317	50	0.309	0.029	50
**Intra-class correlation and inter-rater reliability, all HIATUS Items**	**The Netherlands**			**Germany**		
ICC (SE)	0.026 (0.09)			0.263 (0.27)		
Estimated reliability of mean scores	0.290			0.829		
R-squared	0.06			0.20		
*N*	50			50		

*rho*: Pearson’s rho; *N*: observations/number of items. ICC: estimated from a one-way analysis of variance. SE: standard error. R-squared: coefficient of determination. * No statistical test performed for D2 due to low number of items.

**Table 3:** HIS performance by country and between country difference in performance.

		The Netherlands (NL)			Germany (DE)			Between-Country Gap
		within Country Gap			within Country Gap			
Dimensions	Maximum Achievable Score	Achieved Score	Abs.	%	Achieved Score	Abs.	%	Abs. (NL *minus* DE)
D1—Data sources & availability	39	28	11	28	3	36	92	25
D1.1—Population based records	10	6	4	40	1	9	90	5
D1.2—Health records	8	7	1	13	1	7	88	6
D1.3—Sub-group specific records	8	3	5	63	0	8	100	3
D1.4—Resource records	6	5	1	17	1	5	83	4
D1.5—Microdata	7 *	3.5 *	3.5	50	0 *	7	100	3.5
D2—HIS resources & capacity	8 **	2 **	6	75	2 **	6	75	0
D2.1 Coordination, Planning and Policies	8 **	2 **	33	66	2 **	40	80	0
D3—Published indicators and reports	50	17	6	75	10	7	88	7
D3.1—Self-reported health	8	2	9	75	1	12	100	1
D3.2—Non-communicable diseases	12	3	5	63	0	5	63	3
D3.3—Infectious diseases	8	3	3	38	3	4	50	0
D3.4—Mental health	8	5	5	83	4	4	67	1
D3.5—Socio-economic status	6	1	5	63	2	8	100	−1
D3.6—Health behaviour	8	3	50	52	0	82	85	3
Total HIATUS Score	97	47	11	28	15	36	92	32

* Weighted by the factor 0.5 to put less emphasise on microdata availability; ** Weighted by the factor 2 to compensate for the comparably small number of items in dimension D2. Abs.: Absolute difference. %: Relative difference.
